# Characteristics of Microbiota in Different Segments of the Digestive Tract of Orange‐Collared Keelback (*Rhabdophis himalayanus*)

**DOI:** 10.1002/ece3.74508

**Published:** 2026-10-09

**Authors:** Ji Chen, Jiuyan Jiang, Xia Zhou, Linping Fan, Fei Wu, Pingwei Liang, Huina Song, Ji Wang, Jingxue Luo, Guangxiang Zhu

**Affiliations:** ^1^ College of Life Science Sichuan Agricultural University Ya'an China; ^2^ College of Medical Technology Meishan Pharmaceutical College Meishan China

**Keywords:** evolution, gut microbiota, metabolomics, metagenomics, *Rhabdophis himalayanus*

## Abstract

The gastrointestinal tract of animals plays an essential role in digestion and nutrient absorption. The diversity of gut microbiota is critical for maintaining the physiological functions of animals. However, current research has focused primarily on mammals; research on the gut microbiota of reptiles, particularly snakes, remains limited. This study utilized metagenomic sequencing and untargeted metabolomics to investigate the microbial community composition and functional potential in the stomach, large intestine, and small intestine of the Orange‐collared Keelback ( *Rhabdophis himalayanus* ). The results demonstrate that the three most dominant phyla in the *R. himalayanus* gut microbiota are Pseudomonadota, Actinomycetota, and Bacillota. Moreover, analysis of microbial communities across the stomach, small intestine, and large intestine shows no significant differences in α‐diversity and β‐diversity among the three regions. Linear Discriminant Analysis Effect Size (LEfSe) reveals significant differences in the gut microbiota abundance. MetaStats analysis further reveals significant differences in the abundance of both microbial taxa and CAZymes. Although overall metabolite profiles did not differ significantly among gastrointestinal regions, pairwise analyses identified differentially abundant metabolites associated with amino acid, lipid, nucleotide, and cofactor metabolism. Gut microbiota from different parts of the digestive tract may reveal a unique characteristic of the gut microbiome of *R. himalayanus* and the impact of microbial community structure on function. Collectively, these findings expand our understanding of gastrointestinal microbial communities and associated functional potential in an understudied snake species.

## Graphical abstract

Using shotgun metagenomic sequencing and untargeted metabolomics, we characterized the microbial communities and metabolic profiles across the stomach, small intestine, and large intestine of *Rhabdophis himalayanus* . Although overall microbial diversity was similar among digestive tract segments, distinct taxonomic, functional, and metabolic differences were identified, revealing spatial heterogeneity in the reptilian gut ecosystem. These findings provide new insights into host–microbiome interactions and establish a foundation for future ecological and evolutionary studies of reptilian gut microbiomes.

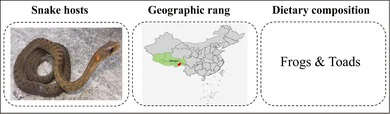

## 1 Introduction

The complex interactions between gut microbiota and their vertebrate hosts, shaped by long‐term coevolutionary processes, play fundamental roles in host health, metabolism, and energy homeostasis (Flint et al. 2012; Jia et al. 2020; Wang et al. 2022; Zhao et al. 2018). The gastrointestinal tract (GIT) consists of multiple compartments, such as the stomach, small intestine, and large intestine, each characterized by distinct physicochemical conditions, which may support region‐specific microbial communities (Donaldson et al. 2016). However, some previous studies have focused on fecal samples, which mainly represent distal gut microbiota and may overlook microbial variation across different GIT regions (Donaldson et al. 2016; Kohl et al. 2017). Therefore, characterizing microbiota from different GIT segments may provide complementary information beyond that obtained from fecal samples. Moreover, this taxonomic gap in GIT microbiome research is enormous: to date, investigations have predominantly focused on mammals (Hu et al. 2022; Zhang et al. 2016), fish (Givens et al. 2015; Li et al. 2024), and birds (Zhang et al. 2021; Zhao et al. 2017).

Snakes represent a diversified vertebrate lineage characterized by physiological adaptations, including digestive plasticity that enables rapid restoration of gastrointestinal function after prolonged fasting (Costello et al. 2010). This remarkable adaptability may be facilitated by gut microbiota‐mediated metabolic plasticity and sustain strict carnivory (Starck and Beese 2001, 2002). However, only a few species of wild snakes have been studied in terms of the gut microbiome, including *Agkistrodon piscivorus* (Colston et al. 2015), *Crotalus horridus* (McLaughlin et al. 2015), *Rhabdophis tigrinus* (Tang, Yang, et al. 2019), and *Rhabdophis subminiatus* (Tang, Zhu, et al. 2019). Recent studies on the reptilian gut microbiota have provided valuable insights into its ecological and evolutionary significance. For instance, a study on *Liolaemus parvus* , *Liolaemus ruibali* , and *Phymaturus williamsi* revealed distinct microbial compositions across different gastrointestinal regions (stomach, hindgut, and small intestine), with particularly pronounced differences between the small intestine and hindgut (Kohl et al. 2017). Similarly, a study on *Lycodon rufozonatus* demonstrates that the stomach exhibits higher microbial diversity in captivity than the small and large intestines, with resident microbes enriched in metabolic pathways related to amino acids, lipids, terpenoids, and polyketides (Shang et al. 2023).

The gut microbiome of animals is important for the conservation and management of endangered species (Song et al. 2024). *Rhabdophis himalayanus* is an oviparous snake belonging to the genus *Rhabdophis* and is distributed in Tibet and Yunnan, with a relatively small population size. Although the mitochondrial genome of *Rhabdophis himalayanus* has been reported (Yang et al. 2019), its gastrointestinal microbiome remains poorly characterized. This species primarily preys on frogs and toads (Yoshida et al. 2020). The samples for this study were collected from Motuo County, Tibet Autonomous Region (Figure 1), a tropical mountainous region on the northern edge of the Himalayas, characterized by over 200 days of annual precipitation, with summer rainfall accounting for more than 50% of the total annual precipitation. These ecological and evolutionary traits make it an interesting species for exploring potential associations between host biology and gastrointestinal microbiota. In this study, we employed metagenomic sequencing and untargeted metabolomics to characterize the structural composition and functional potential of microbial communities across three gastrointestinal segments (stomach, small intestine, and large intestine) of *R. himalayanus* . The limited population distribution and the cryptic habitat preferences of the species pose significant challenges for field sampling. Given the currently available sample size, our study represents an exploratory analysis to characterize its digestive tract microbiome. *Rhabdophis himalayanus* represents an understudied snake species inhabiting complex natural environments, where interactions between hosts and microorganisms may influence nutrient utilization, metabolism, and ecological performance. Our findings enhance our understanding of snake gut microecology and provide novel insights into the gastrointestinal microbial composition and functional potential in snakes.

**FIGURE 1:**
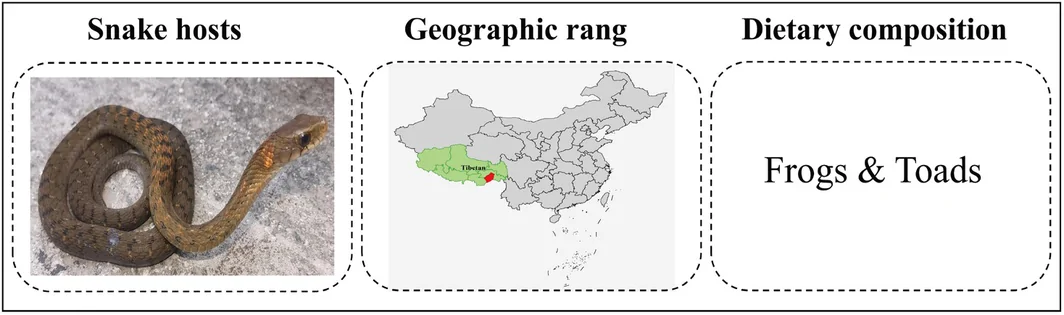
Colour photograph of *R. himalayanus* , its study site, and diet composition. The photograph was taken by the authors.

## 2 Materials and Methods

### 2.1 Sample Collection

Due to challenges in procuring wild individuals, three biological replicates were included in this study. From June to July 2022, we selected and captured three individuals of *R. himalayanus* in Motuo County, located in the southeastern part of the Tibet Autonomous Region. We then placed each animal in a separate sterile, transparent plastic bottle and transported them to the Zoology Laboratory at Sichuan Agricultural University. In this study, the snakes were fasted before sampling, and manual palpation of the abdomen was performed to ensure that no undigested prey remained in the gastrointestinal tract prior to sample collection. We chose the gastrointestinal contents for analysis because they provide a more accurate reflection of gut microbiota than fecal samples. We collected the gastrointestinal contents from each section (RHW‐stomach, RHX‐small intestine, and RHD‐large intestine) into separate sterile 2‐mL collection tubes, which yielded a total of nine samples. We promptly froze the samples in liquid nitrogen for rapid preservation, and we subsequently stored them at −80°C.

### 2.2 DNA Extraction, Metagenomic Sequencing, and Read Preprocessing

Total genomic DNA was extracted from gastrointestinal contents using the Tian‐gen DNA Stool Mini Kit (TIANGEN Biotech, Beijing, China) according to the manufacturer's instructions. We then analyzed the size, concentration, and mass of the nucleic acid fragments using the Agilent 5400 Fragment Analyzer System. Metagenomic shotgun sequencing libraries were constructed and sequenced by Novogene (Beijing, China). After sequencing, quality control was performed. The sequencing reads were then aligned to the reference genome using Bowtie2 software (v2.5.4). Due to the lack of genomic data for *R. himalayanus* , we employed its closely related species, *Rhabdophis nuchalis* (Duan et al. 2024), as a reference for comparison. Reads corresponding to the host genome and low‐quality data were filtered out to obtain clean reads for subsequent analysis (Karlsson et al. 2012, 2013).

### 2.3 Metagenome Assembly, Gene Prediction, and Annotation

Clean data were assembled using MEGAHIT software (v1.2.0) (Li et al. 2015). The resulting scaffolds were segmented at N‐connections to obtain N‐free scaftigs (Li et al. 2015). Next, MetaGeneMark (v3.38) was employed to predict open reading frames (ORFs) from the scaftigs of each sample (Karlsson et al. 2012; Li et al. 2014). ORFs with lengths ≥ 100 nucleotides were initially screened, and CD‐HIT (v4.8.1) software was employed to perform clustering to eliminate redundancy and construct a preliminary non‐redundant gene catalog (Fu et al. 2012; Li et al. 2014). Then, Bowtie2 (v2.5.4) was used to align the high‐quality data from each sample to the non‐redundant gene catalog. Genes with sequencing reads ≥ 3 were selected to form the Unigenes collection (Karlsson et al. 2013; Zeller et al. 2014). Finally, the abundance of each gene in each sample was calculated based on the number of reads mapped to the gene and its length (Buchfink et al. 2015).

### 2.4 Species and Functional Annotation

The DIAMOND software (v2.1.10) was utilized to align Unigenes against microbial sequences in the microNR database (Karlsson et al. 2013; Zeller et al. 2014; Buchfink et al. 2015). For each sequence alignment, we retained results with *e*‐values ≤ 1 × 10^−5^. Since a single sequence might generate multiple matches, we applied the LCA (Lowest Common Ancestor) algorithm to obtain taxonomic abundance profiles at phylum, family, genus, and species levels (Buchfink et al. 2015; Duan et al. 2024; Karlsson et al. 2012; Li et al. 2015). Additionally, DIAMOND was employed to align Unigenes against CAZy and eggNOG for functional annotation, with the highest‐scoring alignment selected for downstream analysis (Buchfink et al. 2015; Cantarel et al. 2009; Huerta‐Cepas et al. 2016; Li et al. 2014).

### 2.5 Metabolomics Analysis

Metabolomic profiling was performed using an Ultra‐high performance liquid chromatography system (Vanquish UHPLC, Thermo Fisher Scientific, Germany) coupled with a Q Exactive HF‐X mass spectrometer (Thermo Fisher Scientific, Germany). Chromatographic separation was achieved using a Hypersil Gold C18 column (100 × 2.1 mm, 1.9 μm). The column temperature was maintained at 40°C with a flow rate of 0.2 mL/min. The mobile phases consisted of 0.1% formic acid in water (A) and methanol (B). Mass spectrometry data were acquired in both positive and negative electrospray ionization modes with a scan range of m/z 100–1500. The spray voltage was set at 3.5 kV, capillary temperature at 320°C, and auxiliary gas heater temperature at 350°C.

Raw LC–MS data files were processed using Compound Discoverer 3.3 (Thermo Fisher Scientific) for peak alignment, peak picking, and metabolite quantification (Souza and Patti 2021). Metabolites were identified by matching retention time, accurate mass, and MS/MS fragmentation spectra against the mzCloud, mzVault, and MassList databases. Background ions were removed using blank samples. Peak intensities were normalized based on total ion intensity, and metabolites with a coefficient of variation (CV) > 30% in QC samples were excluded from further analyses. Differential metabolites were identified based on the PLS‐DA model with VIP > 1, and (FC ≥ 2 or ≤ 0.5), and Student's *t*‐test, *p* < 0.05. The volcano plots were visualized using the R package ggplot2. Identified metabolites were annotated against the KEGG, HMDB, and LIPID MAPS databases (Wishart et al. 2022). KEGG pathway enrichment analysis was performed to identify pathways associated with differentially abundant metabolites. The enrichment results were visualized using the R package ggplot2. Principal component analysis (PCA) was performed using the metaX package in R (Wen et al. 2017).

## 3 Results

### 3.1 Sequencing of the Gastrointestinal Microbiota of *Rhabdophis himalayanus*

A total of nine sets of gastrointestinal sequencing data for *R. himalayanus* were obtained in this study (Table S1). The original data ranged from 6.01 Gb to 6.85 Gb, with an average of 6.58 ± 0.28 Gb; the data after quality control ranged from 5.87 to 6.62 Gb, with an average of 6.39 ± 0.24 Gb; the proportion of high‐quality bases (with quality values ≥ Q20) in the quality‐controlled data was from 97.42% to 97.79%, with an average of 97.58% ± 0.14%; the proportion of high‐quality bases with quality values ≥ Q30 was from 93.56% to 94.39%, with an average of 93.88% ± 0.32%. The GC content of each sample ranged from 41.72% to 42.53%, with an average of 42.20% ± 0.30%; the efficiency (the proportion of valid reads in clean reads) ranged from 95.54% to 98.23%, with an average of 97.05% ± 1.08%.

Venn diagram analysis showed that there were 650,624 overlapping clusters in the three groups, which helped to clarify how the number of genes varied between the groups. In addition, the numbers of gene clusters unique to RHD, RHX, and RHW groups were 2817, 5998, and 3871, respectively (Figure 2), and the number of unique genes in the RHX group was higher than that in the RHD and RHW groups.

**FIGURE 2:**
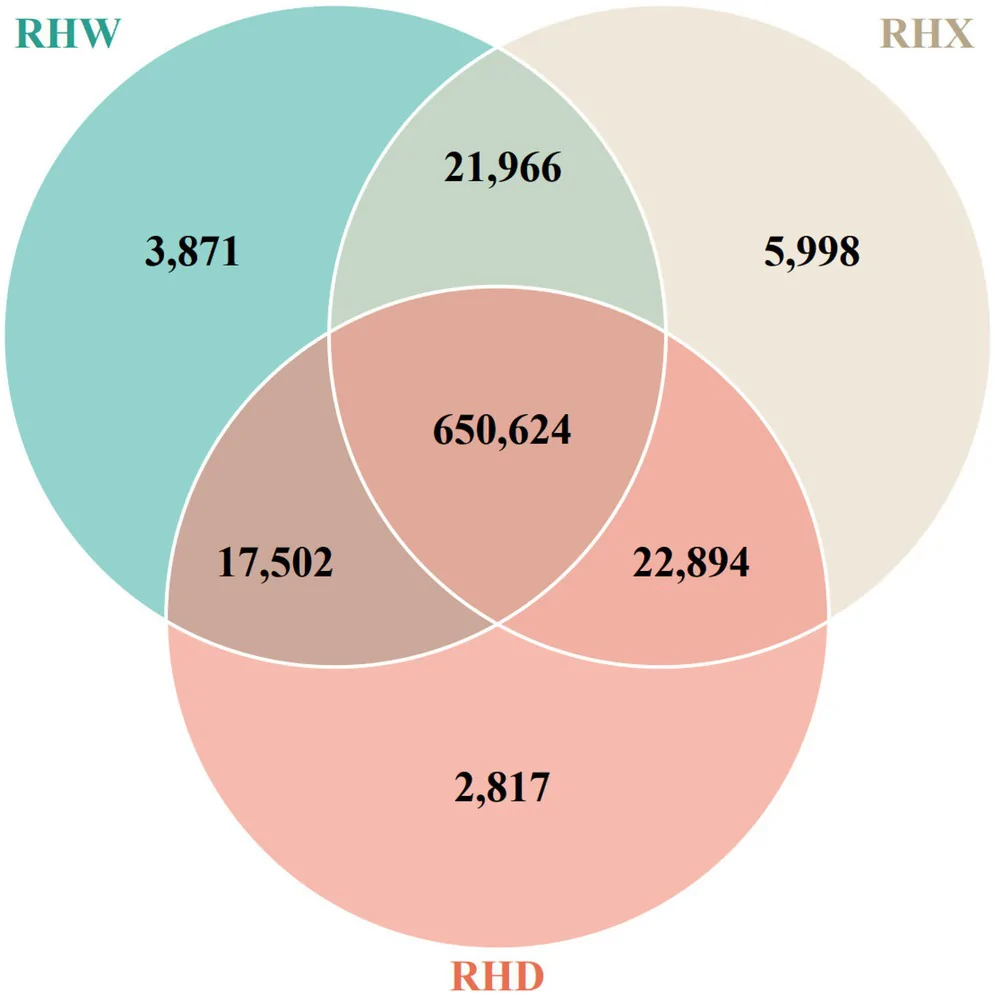
Venn diagram of unique genes in different gastrointestinal segments. RHD, large intestine; RHX, small intestine; RHW, stomach.

### 3.2 Microbial Diversity in the Gastrointestinal Tract of *Rhabdophis himalayanus*

In alpha diversity analysis, Good's coverage index was greater than 99.9%, indicating that the sequencing depth was sufficient to represent the microbial diversity of the samples. In addition, the *p*‐values of the α‐diversity indices were all greater than 0.05, indicating that the α‐diversity did not differ significantly among the stomach, small, and large intestines (Figure 3A, Figure S1).

**FIGURE 3:**
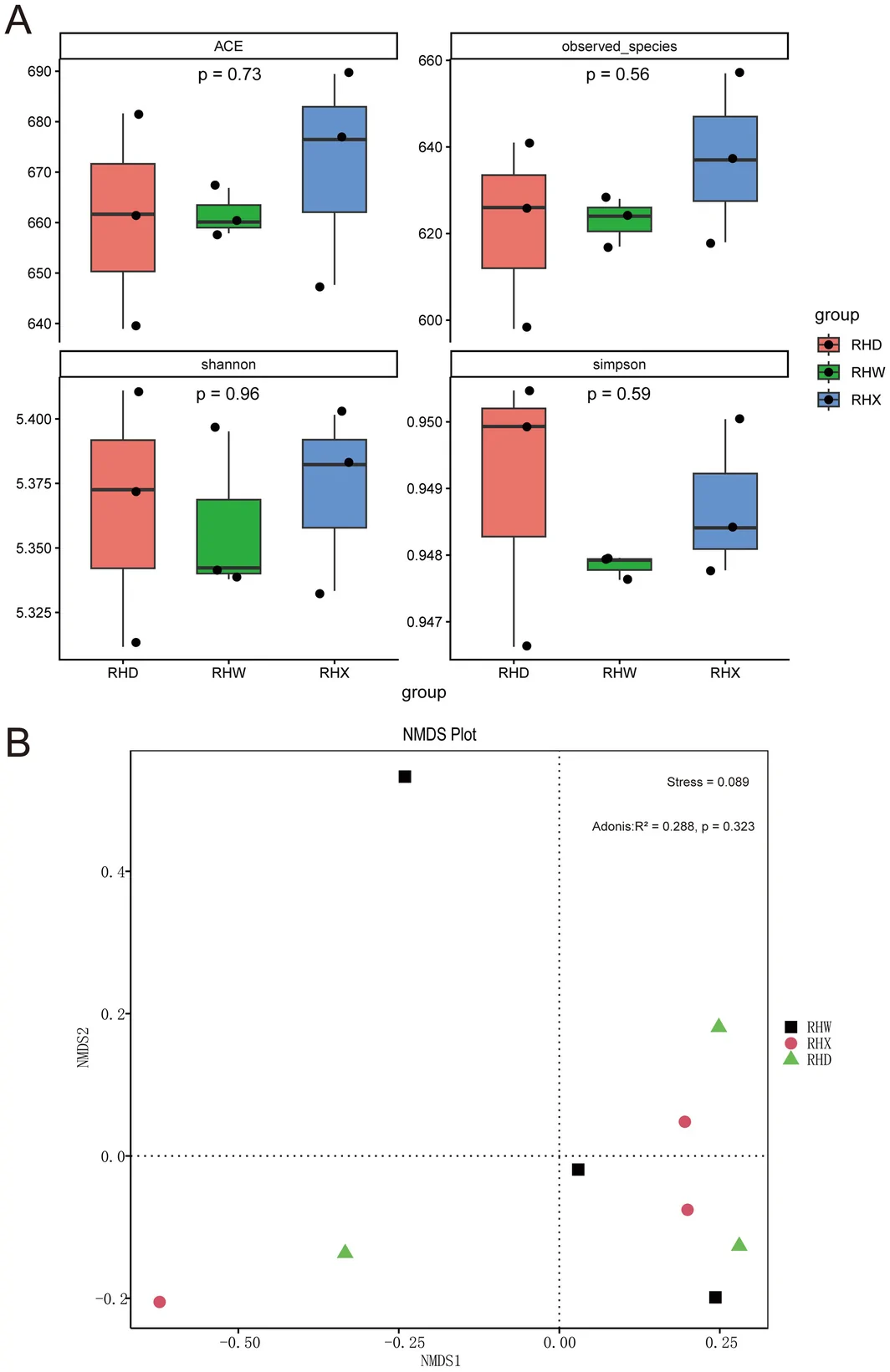
(A) Alpha diversity analysis. The alpha diversity indices include ACE, observed species, Shannon and Simpson. (B) Non‐metric Multidimensional Scaling (NMDS) analysis of gut microbiota distances in RHW, RHX and RHD. RHD, large intestine; RHW, stomach; RHX, small intestine.

The NMDS analysis results, with a stress value of 0.089, indicated that the analysis can reasonably reflect spatial differences. Although some samples showed a certain degree of overlap in their distribution, no significant aggregation was observed (Figure 3B). Adonis (*R* ^2^ = 0.288, *p* = 0.323) showed that there is no significant difference across the different samples.

### 3.3 Comparative Analysis of Microbial Composition and Structure in Different Gastrointestinal Segments

The processed data were compared with those in the microNR database (blastp, *E*‐value ≤ 1 × 10^−5^) to obtain species annotations at different taxonomic levels, obtaining 1462 species belonging to 66 phyla and 774 genera. Taxonomic assignments included bacteria, archaea, eukaryotes, and viruses. Bacteria had the highest abundance in the annotated classifications.

At the phylum level, the top five phyla in terms of relative abundance in the gastrointestinal microbiota were, in order, Pseudomonadota, Actinomycetota, Bacillota, Campylobacterota, and Bacteroidota (Figure 4A). At the genus level, the top five genera in terms of relative abundance in the gastrointestinal microbiota were, in order, *Escherichia*, *Mycobacteroides*, *Campylobacter*, *Nocardia*, and *Thalassococcus* (Figure 4B). At the species level, the top five species in terms of relative abundance in the gastrointestinal microbiota were, in order, *Escherichia coli* , *Mycobacteroides chelonae*, *Campylobacter* sp. CS_ED1, *Thalassococcus profundi*, and *Klebsiella pneumoniae* (Figure 4C). The above data indicate that the stomach, small intestine, and large intestine of *R. himalayanus* shared several dominant microbial taxa.

**FIGURE 4:**
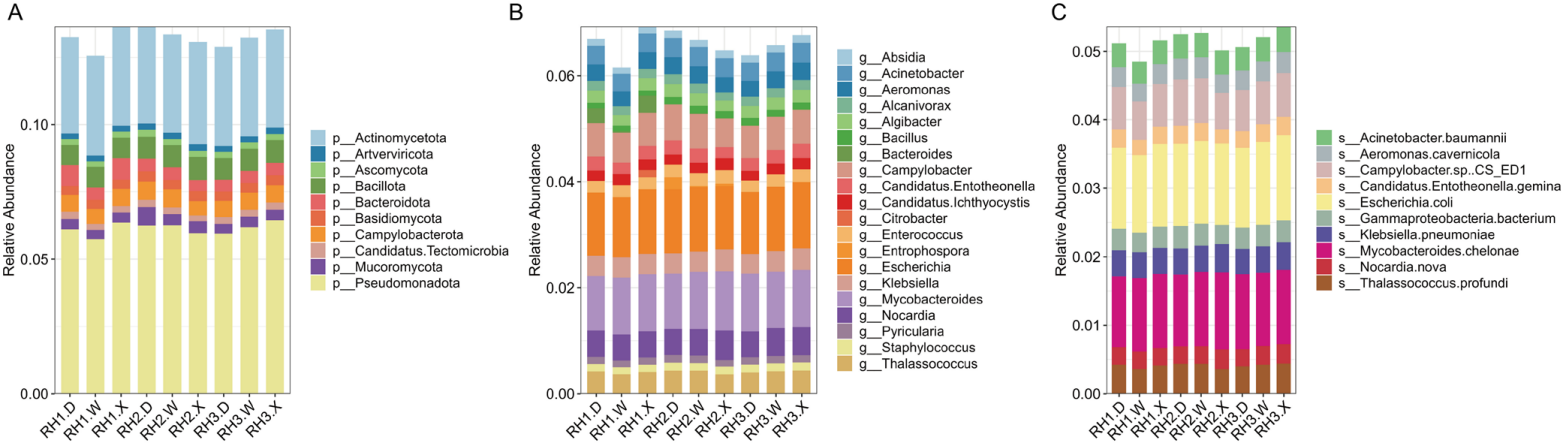
Microbial composition of different samples and gastrointestinal segments at the phylum (A), genus (B) and species (C) levels. RHD, large intestine; RHW, stomach; RHX, small intestine.

A heatmap of the 35 most abundant microbial genera (Figure 5A) revealed that there were 14 microbial genera in the large intestine that showed higher relative abundance (*Aeromonas*, *Alcanivorax*, *Algibacter*, *Bacillus*, *Candidatus Entotheonella*, *Candidatus Nephrothrix*, *Campylobacter*, *Entrophospora*, *Micromonospora*, *Rhizopus*, *Stutzerimonas*, *Thalassococcus*, *Pyricularia*, and *Vibrio*) than in the small intestine. The relative abundance of two genera (*Batrachochytrium*, *Mycobacterium*) was higher in the stomach than in the large intestine. In the small intestine, 19 microbial genera had a higher relative abundance (*Pseudomonas*, *Streptomyces*, *Nocardia*, *Dictyocoela*, *Enterococcus*, *Candidatus Ichthyocystis*, *Parasutterella*, *Stappia*, *Mycobacteroides*, *Shigella*, *Klebsiella*, *Staphylococcus*, *Escherichia*, *Citrobacter*, *Bacteroides*, *Salmonella*, *Absidia*, *Acinetobacter*, *Candidatus Epulonipiscium*) than in the stomach.

**FIGURE 5:**
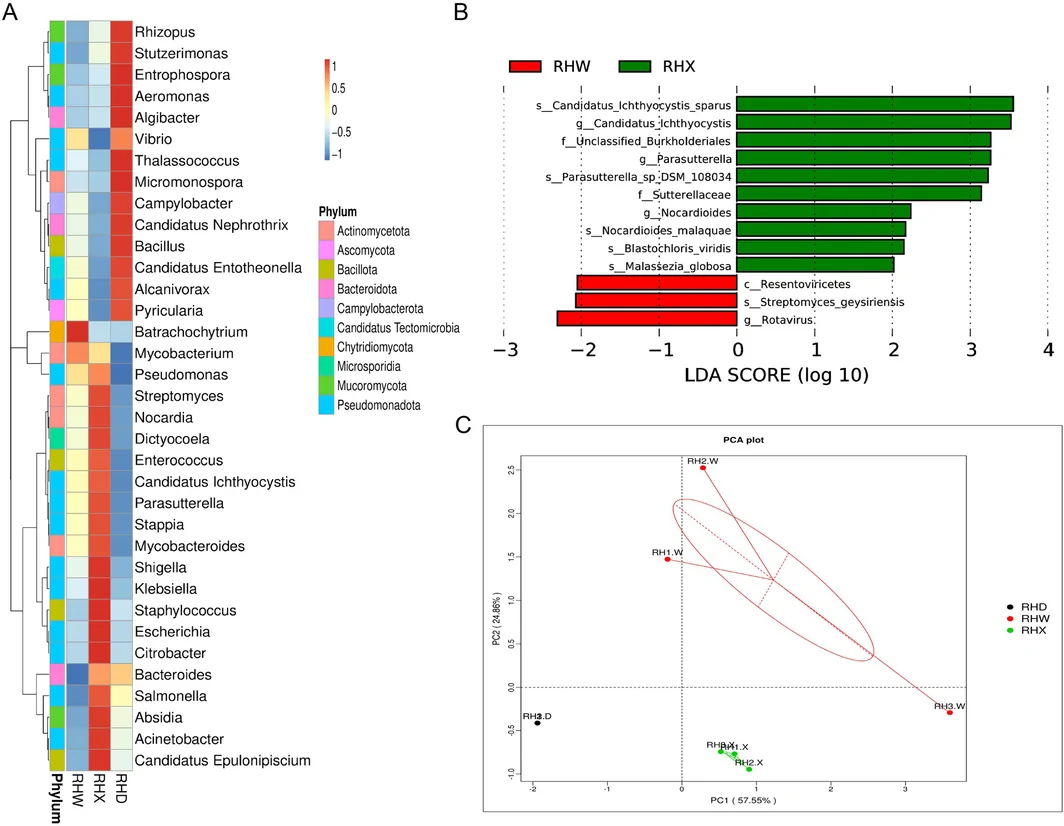
(A) Heatmap displaying the top 35 most abundant genera across experimental groups, highlighting compositional variations at the genus level. (B) Linear Discriminant Analysis Effect Size (LEfSe) analysis was performed to identify features most likely to explain differences between groups. Features with a Kruskal–Wallis test *p* < 0.05 and an LDA score > 2 are shown. The histogram displays features with an LDA score greater than 2, indicating significant differences. (C) Principal component analysis (PCA) ordination plot derived from species‐level microbial community composition based on MetaStats‐identified differential taxa. RHD, large intestine; RHW, stomach; RHX, small intestine.

MetaStats analysis identified differential genera among gastrointestinal regions (*q* < 0.05). The heatmap showed that *Veillonella* had a higher relative abundance in the stomach than in the small intestine. *Trichodelitschia* and *Calothrix* showed the highest relative abundances in the small intestine, followed by the stomach and large intestine. *Stemphylium*, *Sinirhodobacter*, *Pseudohalioglobus*, and *Ramaria* had higher relative abundances in the large intestine than in the stomach. *Candidatus Syntrophoarchaeum*, *Hypsizygus*, and *Beauveria* had higher relative abundances in the small intestine than in the large intestine (Figure S2A). At the species level, *Bacteroides ihuae*, *B. thetaiotaomicron* , *B. fragilis* , *B. ovatus* , *B. xylanisolvens* , *Phocaeicola vulgatus*, *B. graminisolvens* , *B. pyogenes* , *B. salyersiae* , *Fusobacterium varium* , *B. congonensis*, *Caudoviricetes* sp., and *B. cellulosilyticus* were significantly more abundant in the small intestine than in the large intestine; *Xanthomonas citri* and *Pseudomonas monteilii* were significantly more abundant in the large intestine than in the small intestine (Figure S2B).

LEfSe analysis (LDA score > 2, *p* < 0.05) revealed significant microbial disparities between RHW and RHX groups, with RHX exhibiting a greater number of biomarkers (*n* = 10) compared to RHW (*n* = 3). In addition, 10 biomarkers were enriched in RHX while 3 biomarkers were enriched in RHW (Figure 5B). Meanwhile, PCA (Principal Component Analysis) plots were constructed for all the differential microbiota at the species level. The results indicated that the samples from different sites showed a certain separation trend in PCA space (Figure 5C).

### 3.4 Functional Analysis

To better predict the functional potential of the snake gut microbiome, the sequencing data of the snake gut microbiome were subjected to eggNOG functional annotation. It was found that among 24 functional categories, the gut microbiota of wild snakes was predominantly represented by Function unknown (S), Amino acid transport and metabolism (E), Replication, recombination and repair (L), and Carbohydrate transport and metabolism (G) (Figure 6A). Genomic characterization revealed predominant functional annotation among CAZy‐annotated genes, with glycoside hydrolases (GHs) constituting the largest proportion, followed by glycosyltransferases (GTs) and carbohydrate‐binding modules (CBMs) (Figure 6B).

**FIGURE 6:**
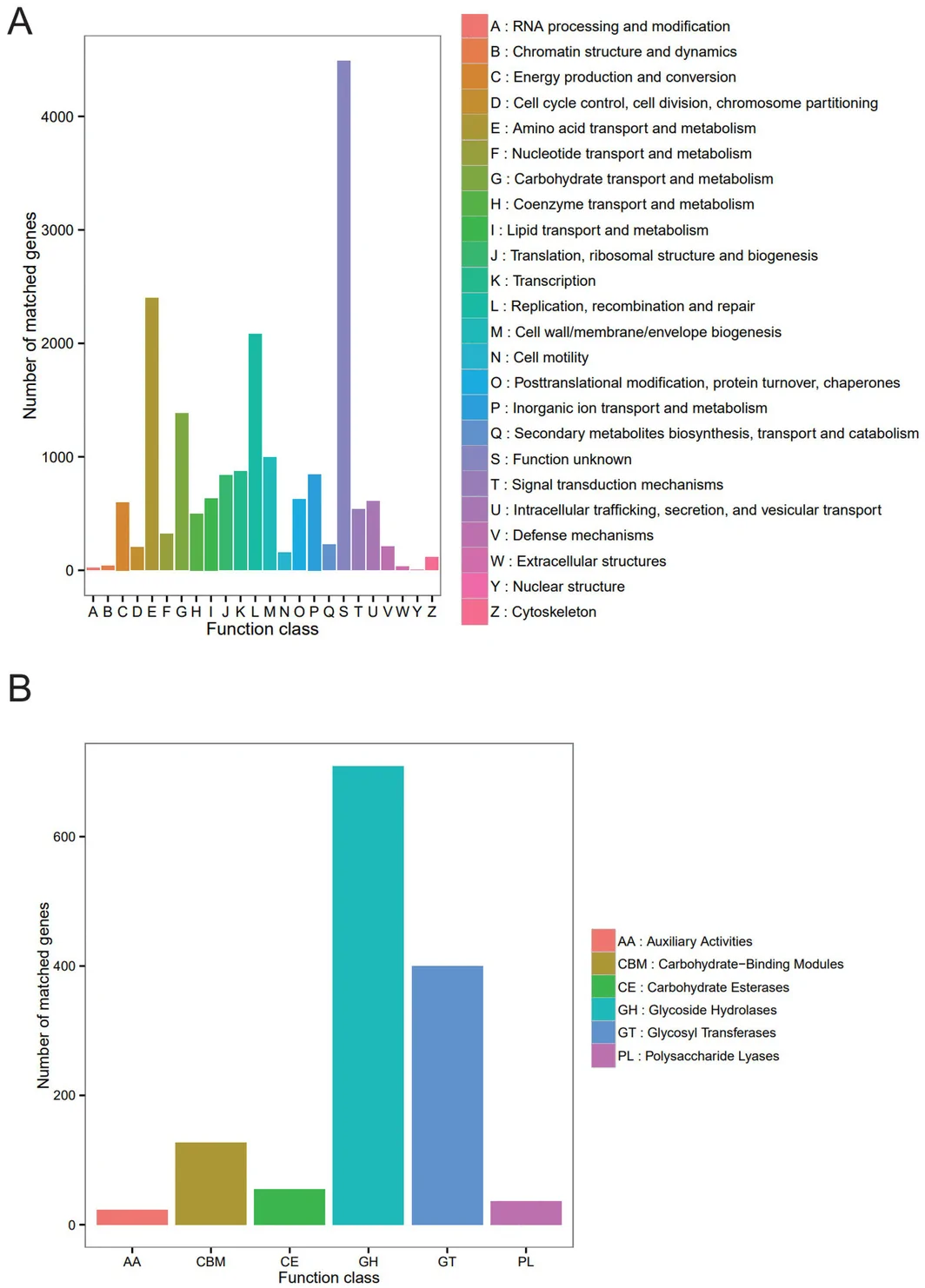
Bar charts illustrate the distribution of functionally annotated genes through (A) the eggNOG (Evolutionary Genealogy of Genes: Non‐supervised Orthologous Groups) database and (B) the CAZy (Carbohydrate‐Active Enzymes) database. RHD, large intestine; RHW, stomach; RHX, small intestine.

At the CAZy family level, CBM14 (Carbohydrate‐Binding Module) and GH18 were the most abundant families. The large intestine had three enzyme families (AA3, GH34, GH146) with higher relative abundance compared to the small intestine. The small intestine exhibited 12 enzyme families (GH2, GH13, GT1, GT4, GT2, GT48, CBM50, CBM18, GH18, CBM14, GH47, GH28) with higher relative abundance than the stomach and large intestine. The stomach and small intestine had five enzyme families (PL4, CE8, GT27, CBM13, GT39) with higher relative abundance compared to the large intestine (Figure S3A). MetaStats was used to compare the relative abundances of CAZy families, and differences in multiple enzymes across different sites were observed. Significant differences were identified in the relative abundances of 12 CAZy families between the small and large intestines (Figure S3B).

### 3.5 Metabolomics Analysis

In the PCA analysis, the first two principal components explained 26.49% and 19.55% of the total variance, respectively. QC samples clustered tightly together, indicating good analytical stability and data reproducibility. PERMANOVA analysis showed that a moderate effect size was observed (*R* ^2^ = 0.351), although the difference among groups did not reach statistical significance (*p* = 0.052). Although partial separation among the three groups was observed, considerable overlap and dispersion within groups were also present, suggesting that the metabolite profiles were not clearly separated among the groups and may exhibit substantial variability among individuals (Figure 7).

**FIGURE 7:**
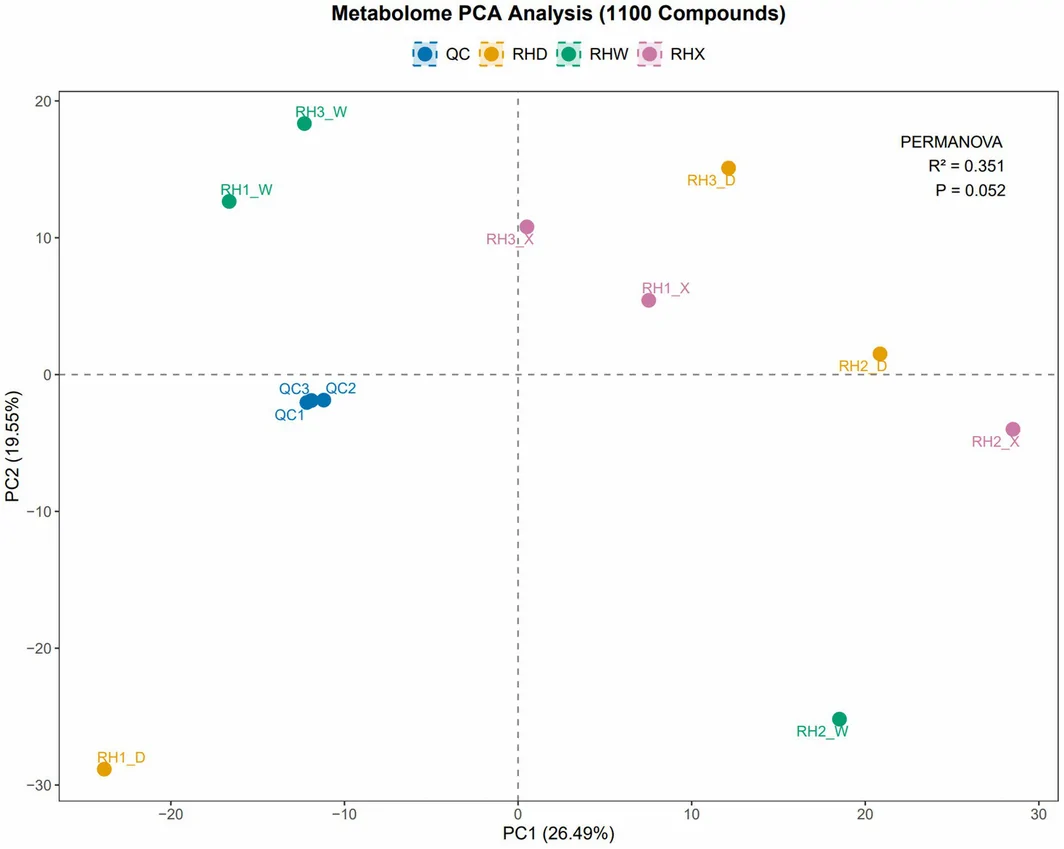
Metabolome PCA analysis. QC, quality control; RHD, large intestine; RHW, stomach; RHX, small intestine.

We used volcano plots to screen for differentially abundant metabolites. The screening criteria included fold change (FC), variable importance in projection (VIP), and *p*‐value. In the positive ion mode, between the RHD and RHX groups, 8 differentially abundant metabolites were identified, with 6 metabolites significantly upregulated and 2 metabolites significantly downregulated. Between the RHW and RHD groups, 27 differentially abundant metabolites were identified, with 7 metabolites significantly upregulated and 20 metabolites significantly downregulated. Between the RHW and RHX groups, 33 differentially abundant metabolites were identified, with 10 metabolites significantly upregulated and 23 metabolites significantly downregulated (Figure 8A–C). In the negative ion mode, between the RHD and RHX groups, 5 differentially abundant metabolites were identified, with 4 metabolites significantly upregulated and 1 metabolite significantly downregulated. Between the RHW and RHD groups, 24 differentially abundant metabolites were identified, with 17 metabolites significantly upregulated and 7 metabolites significantly downregulated. Between the RHW and RHX groups, 18 differentially abundant metabolites were identified, with 10 metabolites significantly upregulated and 8 metabolites significantly downregulated (Figure 8D–F).

**FIGURE 8:**
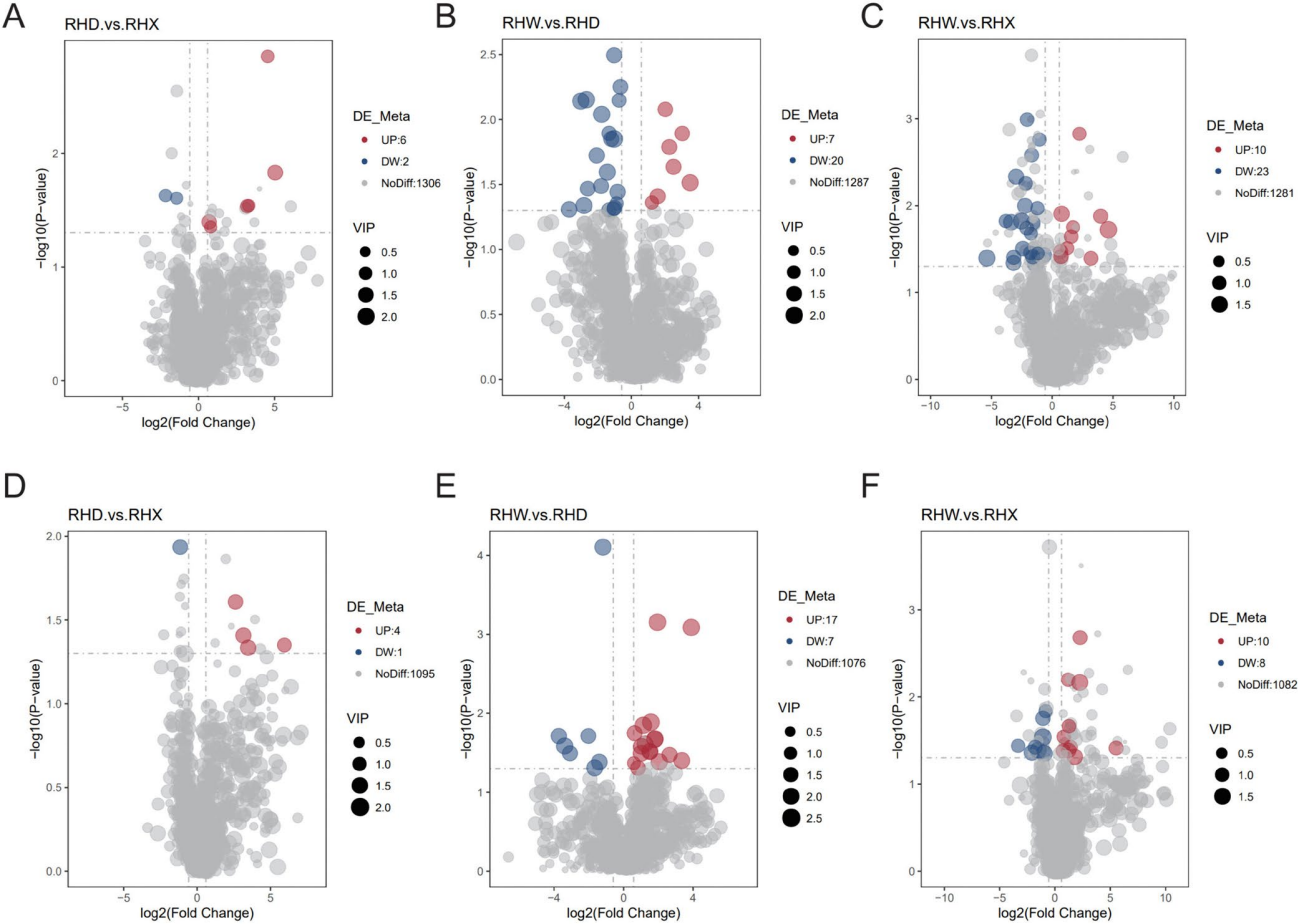
Volcano plots of differential metabolites. Panels A–C show the positive ion mode, and panels D–F show the negative ion mode. The x‐axis represents log_2_(Fold Change), and the y‐axis indicates −log_10_(*p* value). Red dots represent metabolites with significantly higher abundances, blue dots represent metabolites with significantly lower abundances, and gray dots indicate metabolites without significant differences. Bubble size corresponds to the variable importance in projection (VIP) value. RHD, large intestine; RHW, stomach; RHX, small intestine.

KEGG pathway enrichment analysis revealed that the differential metabolites among groups were mainly associated with amino acid metabolism, nucleotide metabolism, lipid metabolism, and cofactor‐related metabolic pathways. In the positive ion mode, between RHD and RHX groups, the differential metabolites were primarily enriched in tryptophan metabolism, histidine metabolism, and β‐alanine metabolism. Between RHW and RHX groups, the differential metabolites were primarily enriched in biosynthesis of unsaturated fatty acids, steroid hormone biosynthesis, pyrimidine metabolism, phenylalanine metabolism, pantothenate and CoA biosynthesis, glycine, serine and threonine metabolism, β‐alanine metabolism, amino sugar and nucleotide sugar metabolism, and α‐linolenic acid metabolism (Figure 9A,B).

**FIGURE 9:**
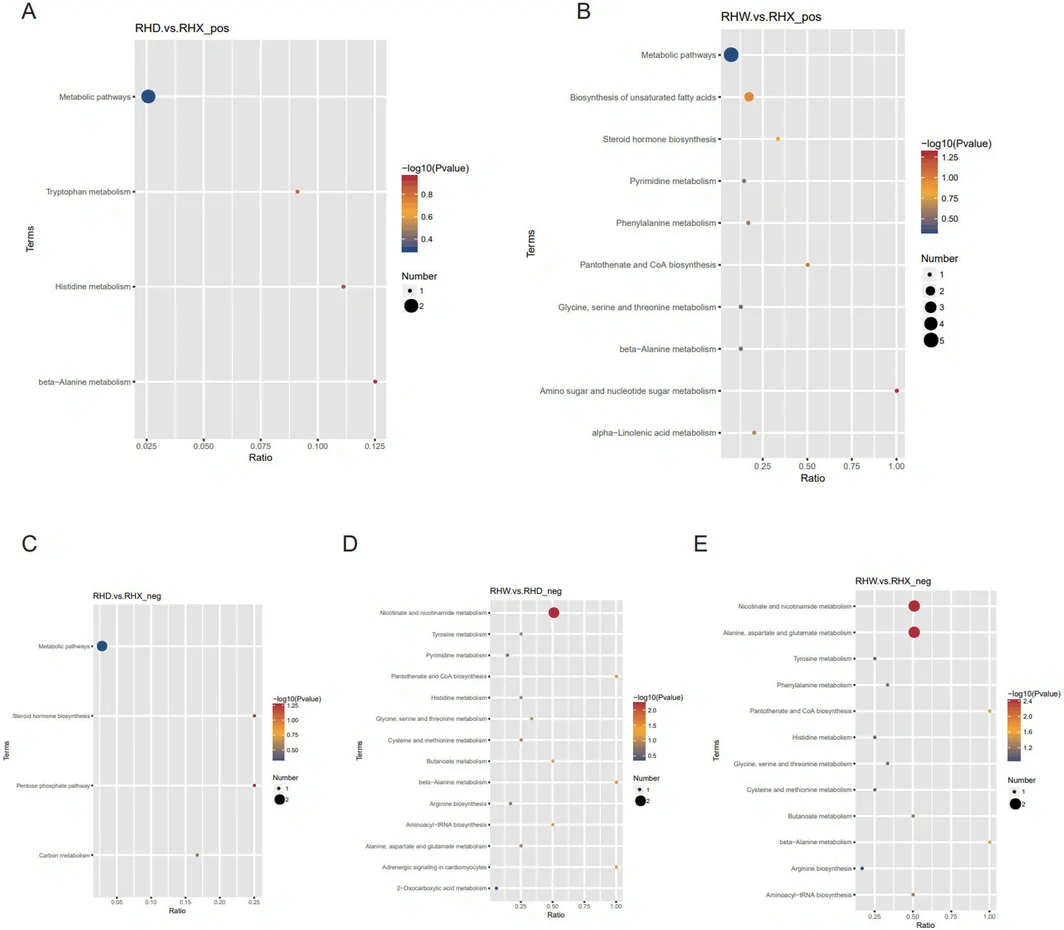
KEGG pathway enrichment analysis of differential metabolites. Panels A–B show the positive ion mode, and panels C–E show the negative ion mode. The x‐axis represents the enrichment ratio, and the y‐axis indicates the enriched KEGG pathways. Bubble size corresponds to the number of differential metabolites enriched in each pathway, and bubble color represents the enrichment significance expressed as −log_10_(*p* value). RHD, large intestine; RHW, stomach; RHX, small intestine.

In the negative ion mode, between RHD and RHX groups, the differential metabolites mainly involved metabolic pathways, steroid hormone biosynthesis, the pentose phosphate pathway, and carbon metabolism. Comparisons of RHW with RHD and RHX demonstrated that the differential metabolites were predominantly enriched in nicotinate and nicotinamide metabolism, tyrosine metabolism, histidine metabolism, glycine, serine and threonine metabolism, cysteine and methionine metabolism, β‐alanine metabolism, arginine biosynthesis, alanine, aspartate and glutamate metabolism, pantothenate and CoA biosynthesis, aminoacyl‐tRNA biosynthesis, and butanoate metabolism (Figure 9C–E).

## 4 Discussion

As obligate carnivores, snakes typically consume high‐protein, low‐carbohydrate diets. Such dietary habits lead to a unique gut microbial community structure and function, adapted to their carnivorous feeding behaviour (Glaudas et al. 2019). Similar to other vertebrates, the composition of the gut microbiota in different parts of the digestive tract plays a significant role in the growth of snakes.

The stomach, small intestine, and large intestine of *R. himalayanus* shared several dominant microbial taxa, with no statistically significant differences detected in microbial diversity. Studies demonstrate that low‐abundance microbiota play critical functional roles, including significant modulation of host immune responses (Han et al. 2022) and regulation of host metabolic processes while maintaining microbial community stability (Benjamino et al. 2018). MetaStats analysis identified differences in the relative abundances of microbial taxa among the stomach, large intestine, and small intestine of *R. himalayanus* . LEfSe analysis further identified several taxa that differed in abundance between the stomach and small intestine, suggesting potential variation in microbial composition between these gastrointestinal regions. Furthermore, *Nocardia* and *Thalassococcus* were detected, which may tentatively suggest that the gastrointestinal microbiota of snakes could be influenced by exogenous environments.

Pseudomonadota has been identified as a dominant phylum in other *Rhabdophis* species, including *R. tigrinus* (Tang, Yang, et al. 2019) and *R. subminiatus* (Tang, Zhu, et al. 2019). This phylum, which includes genera like *Escherichia*, typically mediates complex carbohydrate fermentation and potentially contributes to host vitamin biosynthesis (Colston and Jackson 2016). A study reported that European children typically consume diets rich in fat and protein, which is associated with a high abundance of *Escherichia* (De Filippo et al. 2010). Pseudomonadota has also been reported as a dominant phylum in several other snake species, including *Hydrophis curtus* and *H. cyanocinctus* (Zhong et al. 2022), *Protobothrops mucrosquamatus* , *Elaphe dione* , and *Gloydius angusticeps* (Hu et al. 2024), *Laticauda laticaudata* , *Trimeresurus flavomaculatus* , and *Boiga dendrophila* (Smith et al. 2021), which further suggests that Pseudomonadota may play an important role in the metabolic system of snakes.

Members of Actinomycetota exhibit diverse ecological roles, with some taxa associated with beneficial functions and others reported as opportunistic pathogens under specific conditions (Norouzi et al. 2018). In many mammalian gut microbiome studies, Actinomycetota generally represent a relatively low proportion of the community. However, elevated proportions (> 1%) have been documented in African penguins (Graciette et al. 2023), Stejneger's bamboo pitvipers (Zhang et al. 2024), and *R. himalayanus* . Furthermore, research on the Cottonmouth Snake revealed that increased Pseudomonadota abundance suggests greater similarity to that of birds (Colston et al. 2015). These observations suggest that some aspects of the gut microbiota composition of *R. himalayanus* may share similarities with those reported in birds. Such similarities reflect that host evolutionary history, together with ecological and physiological factors, may be associated with variation in gut microbiota composition across vertebrates.

Comparative analyses of *Viridovipera stejnegeri* populations across geographic regions demonstrated no significant differences in gut microbiota alpha‐diversity, with significant differences observed in the composition and abundance of CAZymes (Zhang et al. 2024). Studies with *Lycodon rufozonatus* and *L. rosozonatus* showed no significant differences in the composition or diversity of the gut microbiota, but differences in the function of carbohydrate enzymes (Zhu et al. 2024). These findings prompt us to further investigate the biological functions of the gut microbiota in *R. himalayanus* .

The enrichment of amino acid and carbohydrate metabolic pathways observed in *R. himalayanus* may contribute to metabolic processes associated with its carnivorous diet and intermittent feeding strategy. These findings provide potential insights into the relationship between gut microbial functions and host dietary ecology. This metabolic specialization aligns with patterns documented in other serpentine species: *Crotalus horridus* (McLaughlin et al. 2015), *Ptyas dhumnades* (Li et al. 2021), *L. rufozonatus* (Shang et al. 2023), *Hydrophis curtus* , and *H. cyanocinctus* (Zhong et al. 2022). The gut microbiota is primarily associated with metabolic pathways, and it not only facilitates the acquisition of energy but also maintains functions related to nutrient metabolism. CAZy annotation further characterized the carbohydrate‐related functional potential of the gastrointestinal microbiome. Among the CAZy‐annotated genes, glycoside hydrolases (GHs) accounted for the largest proportion, consistent with findings reported for *L. rufozonatus* and *L. rosozonatus* (Zhu et al. 2024). Notably, the high relative abundances of CBM14 and GH18 annotations suggest a potential capacity for chitin binding and degradation, given the chitin‐binding functions of CBM14 modules and the occurrence of chitinases within the GH18 family (Funkhouser and Aronson 2007; Huang et al. 2012). Ecologically, *R. himalayanus* mainly preys on frogs and toads. Because anuran prey may contain chitinous material derived from ingested arthropods, such dietary components could potentially reach the snake's gastrointestinal tract and provide substrates for microbial degradation.

Although overall metabolite profiles did not differ significantly among gastrointestinal regions, pairwise comparisons identified differences in individual metabolites. These metabolites mapped to pathways associated with amino acid, lipid, nucleotide, and cofactor metabolism. In mammals, the colonic segment of the large intestine is a typical anaerobic fermentation environment where anaerobic microorganisms selectively colonize, which together contribute to elevated metabolic activity and functional potential (Blaut and Clavel 2007). In the stomach, despite the harsh acidic environment, gastric microbiota can exhibit adaptive metabolic responses to low pH and oxidative stress, thereby contributing to active microbial metabolic functions and metabolite production (Suzuki et al. 2012; Dai et al. 2021; Santacroce et al. 2024). Notably, the relatively lower abundance of metabolism‐related pathways in the small intestine may be associated with its unique physicochemical environment. The flow rate of the small intestine's contents is relatively fast, making it difficult for microorganisms to form dense, stable colonies (Kastl et al. 2020). Compared to the large intestine, the small intestine has a higher oxygen content, which limits the colonization of anaerobic microorganisms and the occurrence of fermentation (Wang et al. 2024). These findings suggest that the structural specificity of the small intestine limits the complexity of microbial composition and function compared to the large intestine and stomach. In KEGG enrichment analysis, differential metabolites were mainly associated with amino acid metabolism, lipid metabolism, nucleotide metabolism, and cofactor biosynthesis pathways, suggesting substantial metabolic reprogramming under different physiological conditions. Differentially abundant metabolites mapped to several amino acid‐related pathways, including tryptophan metabolism and phenylalanine metabolism. Previous studies have demonstrated that amino acid metabolism plays a crucial role in host immune regulation and gut microbial interactions (Liu et al. 2020). In particular, tryptophan metabolism has emerged as a key regulatory axis mediating microbiota–host crosstalk, as microbially derived tryptophan metabolites can modulate gastrointestinal homeostasis, mucosal barrier integrity, and inflammatory responses through activation of signaling pathways such as the aryl hydrocarbon receptor (AHR) (Agus et al. 2018; Wang et al. 2024). In addition, alterations in aromatic amino acid metabolism, including tryptophan and phenylalanine pathways, are closely associated with microbial functional activity and immune homeostasis (Liu et al. 2020; Seo and Kwon 2023). Although our study did not directly evaluate these physiological functions, the observed differences in amino acid‐related metabolic pathways may reflect variation in microbial metabolic potential among the gastrointestinal regions examined.

Our study has several limitations. Although the integration of metagenomic and metabolomic approaches allowed us to characterize the taxonomic composition, functional potential, and metabolic characteristics of the gut microbiota across different digestive tract segments of *R. himalayanus* , the relatively limited sample size may restrict the statistical power and the extent to which our findings can be generalized to broader populations. This limitation mainly resulted from the challenges associated with sampling wild snake species, including difficulties in field collection, habitat accessibility, and ethical considerations. Therefore, our results should be regarded as an initial exploration of digestive tract microbiome organization and host–microbe metabolic interactions in this understudied snake species. Future studies incorporating larger sample sizes, long‐term monitoring, and populations from different geographic regions will be necessary to evaluate the stability and ecological drivers of gut microbiome variation in *R. himalayanus* .

## 5 Conclusion

This exploratory study characterized gastrointestinal microbial communities, metagenomic functional potential, and metabolite profiles in three individuals of *Rhabdophis himalayanus* . LEfSe and MetaStats analyses identified differences in microbial community composition among distinct gastrointestinal regions. In addition, MetaStats analysis revealed variation in relative abundances of CAZy families across different gastrointestinal regions. Metabolomic analyses further indicated that differential metabolites were associated with pathways involved in amino acid, lipid, nucleotide, and cofactor metabolism. Overall, these findings characterize the taxonomic composition and functional potential of the gut microbiota across gastrointestinal regions of *R. himalayanus* , together with the metabolite profiles of gastrointestinal contents. This study expands the available information on the gastrointestinal microbiome of this understudied snake species and provides a reference for future research on microbial communities and digestive physiology in snakes.

## Author Contributions

**Ji Chen:** data curation (equal), formal analysis (equal), visualization (equal), writing – original draft (equal), writing – review and editing (equal). **Jiuyan Jiang:** data curation (equal), formal analysis (equal), investigation (equal), validation (equal), writing – original draft (equal). **Xia Zhou:** formal analysis (equal), investigation (equal), resources (equal). **Linping Fan:** formal analysis (equal), investigation (equal), resources (equal). **Fei Wu:** data curation (equal), formal analysis (equal), investigation (equal). **Pingwei Liang:** data curation (equal), investigation (equal), validation (equal). **Huina Song:** investigation (equal), resources (equal), validation (equal). **Ji Wang:** investigation (equal), methodology (equal), validation (equal). **Jingxue Luo:** formal analysis (equal), investigation (equal), validation (equal). **Guangxiang Zhu:** conceptualization (equal), funding acquisition (equal), project administration (equal), supervision (equal), writing – review and editing (equal).

## Funding

This research was supported in part by a grant to Zhu GX (2025ZNSFSC0280) from the Natural Science Foundation of Sichuan Province.

## Ethics Statement

Snakes were collected and euthanized according to IACUC protocols approved by the Institutional Animal Care and Use Committee of the Sichuan Agricultural University under Approval No. 20210071. The euthanasia of the animals took their welfare into full account. This study did not involve any endangered animals.

## Conflicts of Interest

The authors declare no conflicts of interest.

## Supporting information


**Table S1:** Statistical Information Sheet on Quality Control of Metagenomic Data.
**Figure S1:** Kruskal–Wallis (KW) analysis of the alpha diversity of gut microbiota in three groups. RHD, large intestine; RHX, small intestine; RHW, stomach; ns: p > 0.05.
**Figure S2:** Differential abundance heatmap illustrating the top 15 genera (A) and species (B) with significant inter‐group disparities identified via MetaStats analysis. RHD, large intestine; RHX, small intestine; RHW, stomach.
**Figure S3:** (A) Heatmap delineating inter‐group compositional disparities among the top 15 most prevalent CAZy families at Level 2 taxonomic hierarchy. (B) Differential abundance heatmap of the top 12 enzyme families with significant inter‐group variations identified through MetaStats analysis, visualized at the CAZy Level 2 classification. RHD, large intestine; RHX, small intestine; RHW, stomach.

## Data Availability

The metagenomic sequencing data generated in this study have been deposited in the National Center for Biotechnology Information (NCBI) Sequence Read Archive (SRA) at https://www.ncbi.nlm.nih.gov/bioproject/PRJNA1224950 under BioProject accession number PRJNA1224950. The metabolomics data have been deposited in the National Genomics Data Center (NGDC) OMIX repository at https://ngdc.cncb.ac.cn/omix/release/OMIX018407 under accession number OMIX018407.

## References

[ece374508-bib-0001] Agus, A. , J. Planchais , and H. Sokol . 2018. “Gut Microbiota Regulation of Tryptophan Metabolism in Health and Disease.” Cell Host & Microbe 23: 716–724. 10.1016/j.chom.2018.05.003 29902437

[ece374508-bib-0002] Benjamino, J. , S. Lincoln , R. Srivastava , and J. Graf . 2018. “Low‐Abundant Bacteria Drive Compositional Changes in the Gut Microbiota After Dietary Alteration.” Microbiome 6: 86. 10.1186/s40168-018-0469-5 PMC5944116 29747692

[ece374508-bib-0003] Blaut, M. , and T. Clavel . 2007. “Metabolic Diversity of the Intestinal Microbiota: Implications for Health and Disease.” Journal of Nutrition 137: 751S–755S. 10.1093/jn/137.3.751S 17311972

[ece374508-bib-0004] Buchfink, B. , C. Xie , and D. H. Huson . 2015. “Fast and Sensitive Protein Alignment Using DIAMOND.” Nature Methods 12: 59–60. 10.1038/nmeth.3176 25402007

[ece374508-bib-0005] Cantarel, B. L. , P. M. Coutinho , C. Rancurel , T. Bernard , V. Lombard , and B. Henrissat . 2009. “The Carbohydrate‐Active EnZymes Database (CAZy): An Expert Resource for Glycogenomics.” Nucleic Acids Research 37: D233–D238. 10.1093/nar/gkn663 PMC2686590 18838391

[ece374508-bib-0006] Colston, T. J. , and C. R. Jackson . 2016. “Microbiome Evolution Along Divergent Branches of the Vertebrate Tree of Life: What Is Known and Unknown.” Molecular Ecology 25: 3776–3800. 10.1111/mec.13730 27297628

[ece374508-bib-0007] Colston, T. J. , B. P. Noonan , and C. R. Jackson . 2015. “Phylogenetic Analysis of Bacterial Communities in Different Regions of the Gastrointestinal Tract of *Agkistrodon piscivorus* , the Cottonmouth Snake.” PLoS One 10: e0128793. 10.1371/journal.pone.0128793 PMC4454441 26039313

[ece374508-bib-0008] Costello, E. K. , J. I. Gordon , S. M. Secor , and R. Knight . 2010. “Postprandial Remodeling of the Gut Microbiota in Burmese Pythons.” ISME Journal 4: 1375–1385. 10.1038/ismej.2010.71 PMC3923499 20520652

[ece374508-bib-0009] Dai, D. , Y. Yang , J. Yu , et al. 2021. “Interactions Between Gastric Microbiota and Metabolites in Gastric Cancer.” Cell Death & Disease 12: 1104. 10.1038/s41419-021-04396-y PMC8613192 34819503

[ece374508-bib-0010] De Filippo, C. , D. Cavalieri , M. Di Paola , et al. 2010. “Impact of Diet in Shaping Gut Microbiota Revealed by a Comparative Study in Children From Europe and Rural Africa.” Proceedings of the National Academy of Sciences of the United States of America 107: 14691–14696. 10.1073/pnas.1005963107 PMC2930426 20679230

[ece374508-bib-0011] Donaldson, G. P. , S. M. Lee , and S. K. Mazmanian . 2016. “Gut Biogeography of the Bacterial Microbiota.” Nature Reviews. Microbiology 14: 20–32. 10.1038/nrmicro3552 PMC4837114 26499895

[ece374508-bib-0012] Duan, M. , S. Yang , X. Li , et al. 2024. “Chromosome‐Level Genome Assembly and Annotation of the *Rhabdophis nuchalis* (Hubei Keelback).” Sci Data 11: 850. 10.1038/s41597-024-03708-z PMC11310211 39117633

[ece374508-bib-0013] Flint, H. J. , K. P. Scott , P. Louis , and S. H. Duncan . 2012. “The Role of the Gut Microbiota in Nutrition and Health.” Nature Reviews. Gastroenterology & Hepatology 9: 577–589. 10.1038/nrgastro.2012.156 22945443

[ece374508-bib-0014] Fu, L. , B. Niu , Z. Zhu , S. Wu , and W. Li . 2012. “CD‐HIT: Accelerated for Clustering the Next‐Generation Sequencing Data.” Bioinformatics 28: 3150–3152. 10.1093/bioinformatics/bts565 PMC3516142 23060610

[ece374508-bib-0015] Funkhouser, J. D. , and N. N. Aronson . 2007. “Chitinase Family GH18: Evolutionary Insights From the Genomic History of a Diverse Protein Family.” BMC Evolutionary Biology 7: 96. 10.1186/1471-2148-7-96 PMC1945033 17594485

[ece374508-bib-0016] Givens, C. , B. Ransom , N. Bano , and J. Hollibaugh . 2015. “Comparison of the Gut Microbiomes of 12 Bony Fish and 3 Shark Species.” Marine Ecology Progress Series 518: 209–223.

[ece374508-bib-0017] Glaudas, X. , K. L. Glennon , M. Martins , et al. 2019. “Foraging Mode, Relative Prey Size and Diet Breadth: A Phylogenetically Explicit Analysis of Snake Feeding Ecology.” Journal of Animal Ecology 88: 757–767. 10.1111/1365-2656.12972 30828806

[ece374508-bib-0018] Graciette, A. G. C. , L. A. Hoopes , T. Clauss , F. J. Stewart , and Z. A. Pratte . 2023. “The Microbiome of African Penguins ( *Spheniscus demersus* ) Under Managed Care Resembles That of Wild Marine Mammals and Birds.” Scientific Reports 13: 16679. 10.1038/s41598-023-43899-w PMC10551019 37794122

[ece374508-bib-0019] Han, G. , H. Luong , and S. Vaishnava . 2022. “Low Abundance Members of the Gut Microbiome Exhibit High Immunogenicity.” Gut Microbes 14: 2104086. 10.1080/19490976.2022.2104086 PMC9331198 35881003

[ece374508-bib-0020] Hu, X. , Y. Wei , T. Zhang , et al. 2022. “Gastrointestinal Biogeography of Luminal Microbiota and Short‐Chain Fatty Acids in Sika Deer ( *Cervus nippon* ).” Applied and Environmental Microbiology 88: e0049922. 10.1128/aem.00499-22 PMC9469704 35950850

[ece374508-bib-0021] Hu, X. , L. Yang , Y. Zhang , et al. 2024. “Fecal and Oral Microbiome Analysis of Snakes From China Reveals a Novel Natural Emerging Disease Reservoir.” Frontiers in Microbiology 14: 1339188. 10.3389/fmicb.2023.1339188 PMC10808610 38274764

[ece374508-bib-0022] Huang, Q.‐S. , X.‐L. Xie , G. Liang , et al. 2012. “The GH18 Family of Chitinases: Their Domain Architectures, Functions and Evolutions.” Glycobiology 22: 23–34. 10.1093/glycob/cwr092 21750098

[ece374508-bib-0023] Huerta‐Cepas, J. , D. Szklarczyk , K. Forslund , et al. 2016. “eggNOG 4.5: A Hierarchical Orthology Framework With Improved Functional Annotations for Eukaryotic, Prokaryotic and Viral Sequences.” Nucleic Acids Research 44: D286–D293. 10.1093/nar/gkv1248 PMC4702882 26582926

[ece374508-bib-0024] Jia, Z. , X. Zhao , X. Liu , et al. 2020. “Impacts of the Plateau Environment on the Gut Microbiota and Blood Clinical Indexes in Han and Tibetan Individuals.” mSystems 5: e00660‐19. 10.1128/mSystems.00660-19 PMC6977073 31964769

[ece374508-bib-0025] Karlsson, F. H. , F. Fåk , I. Nookaew , et al. 2012. “Symptomatic Atherosclerosis Is Associated With an Altered Gut Metagenome.” Nature Communications 3: 1245. 10.1038/ncomms2266 PMC3538954 23212374

[ece374508-bib-0026] Karlsson, F. H. , V. Tremaroli , I. Nookaew , et al. 2013. “Gut Metagenome in European Women With Normal, Impaired and Diabetic Glucose Control.” Nature 498: 99–103. 10.1038/nature12198 23719380

[ece374508-bib-0027] Kastl, A. J. , N. A. Terry , G. D. Wu , and L. G. Albenberg . 2020. “The Structure and Function of the Human Small Intestinal Microbiota: Current Understanding and Future Directions.” Cellular and Molecular Gastroenterology and Hepatology 9: 33–45. 10.1016/j.jcmgh.2019.07.006 PMC6881639 31344510

[ece374508-bib-0028] Kohl, K. D. , A. Brun , M. Magallanes , et al. 2017. “Gut Microbial Ecology of Lizards: Insights Into Diversity in the Wild, Effects of Captivity, Variation Across Gut Regions and Transmission.” Molecular Ecology 26: 1175–1189. 10.1111/mec.13921 27862531

[ece374508-bib-0029] Li, D. , C.‐M. Liu , R. Luo , K. Sadakane , and T.‐W. Lam . 2015. “MEGAHIT: An Ultra‐Fast Single‐Node Solution for Large and Complex Metagenomics Assembly via Succinct de Bruijn Graph.” Bioinformatics 31: 1674–1676. 10.1093/bioinformatics/btv033 25609793

[ece374508-bib-0030] Li, D. , Y. Sun , and F. Xu . 2021. “Differences in Faeces Microbiome Composition and Characteristics Between Two Populations of *Ptyas dhumnades* .” Dongwuxue Zazhi 56: 696–706.

[ece374508-bib-0031] Li, J. , H. Jia , X. Cai , et al. 2014. “An Integrated Catalog of Reference Genes in the Human Gut Microbiome.” Nature Biotechnology 32: 834–841. 10.1038/nbt.2942 24997786

[ece374508-bib-0032] Li, M. , H. Liang , H. Yang , et al. 2024. “Deciphering the Gut Microbiome of Grass Carp Through Multi‐Omics Approach.” Microbiome 12: 2. 10.1186/s40168-023-01715-7 PMC10763231 38167330

[ece374508-bib-0033] Liu, Y. , Y. Hou , G. Wang , X. Zheng , and H. Hao . 2020. “Gut Microbial Metabolites of Aromatic Amino Acids as Signals in Host‐Microbe Interplay.” Trends in Endocrinology and Metabolism 31: 818–834. 10.1016/j.tem.2020.02.012 32284282

[ece374508-bib-0034] McLaughlin, R. W. , P. A. Cochran , and S. E. Dowd . 2015. “Metagenomic Analysis of the Gut Microbiota of the Timber Rattlesnake, *Crotalus horridus* .” Molecular Biology Reports 42: 1187–1195. 10.1007/s11033-015-3854-1 25663091

[ece374508-bib-0035] Norouzi, H. , A. Danesh , M. Mohseni , and M. Rabbani Khorasgani . 2018. “Marine Actinomycetes With Probiotic Potential and Bioactivity Against Multidrug‐Resistant Bacteria.” International Journal of Molecular and Cellular Medicine 7: 44–52. 10.22088/IJMCM.BUMS.7.1.44 PMC6134418 30234072

[ece374508-bib-0036] Santacroce, L. , S. Topi , L. Bottalico , I. A. Charitos , and E. Jirillo . 2024. “Current Knowledge About Gastric Microbiota With Special Emphasis on *Helicobacter pylori* ‐Related Gastric Conditions.” Current Issues in Molecular Biology 46: 4991–5009. 10.3390/cimb46050299 PMC11119845 38785567

[ece374508-bib-0037] Seo, S.‐K. , and B. Kwon . 2023. “Immune Regulation Through Tryptophan Metabolism.” Experimental & Molecular Medicine 55: 1371–1379. 10.1038/s12276-023-01028-7 PMC10394086 37394584

[ece374508-bib-0038] Shang, Y. , H. Zhong , G. Liu , et al. 2023. “Characteristics of Microbiota in Different Segments of the Digestive Tract of *Lycodon rufozonatus* .” Animals 13: 731. 10.3390/ani13040731 PMC9952230 36830518

[ece374508-bib-0039] Smith, S. N. , T. J. Colston , and C. D. Siler . 2021. “Venomous Snakes Reveal Ecological and Phylogenetic Factors Influencing Variation in Gut and Oral Microbiomes.” Frontiers in Microbiology 12: 657754. 10.3389/fmicb.2021.657754 PMC8032887 33841384

[ece374508-bib-0040] Song, P. , F. Jiang , D. Liu , et al. 2024. “Gut Microbiota Non‐Convergence and Adaptations in Sympatric Tibetan and Przewalski's Gazelles.” iScience 27: 109117. 10.1016/j.isci.2024.109117 PMC10879710 38384851

[ece374508-bib-0041] Souza, A. L. , and G. J. Patti . 2021. “A Protocol for Untargeted Metabolomic Analysis: From Sample Preparation to Data Processing.” Methods in Molecular Biology 2276: 357–382. 10.1007/978-1-0716-1266-8_27 PMC9284939 34060055

[ece374508-bib-0042] Starck, J. M. , and K. Beese . 2001. “Structural Flexibility of the Intestine of Burmese Python in Response to Feeding.” Journal of Experimental Biology 204: 325–335. 10.1242/jeb.204.2.325 11136618

[ece374508-bib-0043] Starck, J. M. , and K. Beese . 2002. “Structural Flexibility of the Small Intestine and Liver of Garter Snakes in Response to Feeding and Fasting.” Journal of Experimental Biology 205: 1377–1388. 10.1242/jeb.205.10.1377 11976350

[ece374508-bib-0044] Suzuki, H. , T. Nishizawa , H. Tsugawa , S. Mogami , and T. Hibi . 2012. “Roles of Oxidative Stress in Stomach Disorders.” Journal of Clinical Biochemistry and Nutrition 50: 35–39. 10.3164/jcbn.11-115SR PMC3246180 22247598

[ece374508-bib-0045] Tang, W. , S. Yang , Y. Cheng , Q. Shi , C. Zhang , and G. X. Zhu . 2019. “Intestine Microflora Composition and Distribution Characteristics in *Rhabdophis tigrinus* (Squamata: Colubridae).” Chinese Journal of Zoology 54: 589–598.

[ece374508-bib-0046] Tang, W. , G. Zhu , Q. Shi , et al. 2019. “Characterizing the Microbiota in Gastrointestinal Tract Segments of *Rhabdophis subminiatus* : Dynamic Changes and Functional Predictions.” MicrobiologyOpen 8: e00789. 10.1002/mbo3.789 PMC6612554 30848054

[ece374508-bib-0047] Wang, T. , R. X. Wang , and S. P. Colgan . 2024. “Physiologic Hypoxia in the Intestinal Mucosa: A Central Role for Short‐Chain Fatty Acids.” American Journal of Physiology. Cell Physiology 327: C1087–C1093. 10.1152/ajpcell.00472.2024 PMC11482044 39159391

[ece374508-bib-0048] Wang, X. , X. Wu , Y. Shang , et al. 2022. “High‐Altitude Drives the Convergent Evolution of Alpha Diversity and Indicator Microbiota in the Gut Microbiomes of Ungulates.” Frontiers in Microbiology 13: 953234. 10.3389/fmicb.2022.953234 PMC9301279 35875556

[ece374508-bib-0049] Wen, B. , Z. Mei , C. Zeng , and S. Liu . 2017. “metaX: A Flexible and Comprehensive Software for Processing Metabolomics Data.” BMC Bioinformatics 18: 183. 10.1186/s12859-017-1579-y PMC5361702 28327092

[ece374508-bib-0050] Wishart, D. S. , A. Guo , E. Oler , et al. 2022. “HMDB 5.0: The Human Metabolome Database for 2022.” Nucleic Acids Research 50: D622–D631. 10.1093/nar/gkab1062 PMC8728138 34986597

[ece374508-bib-0051] Yang, S. , Q. Shi , W. Tang , C. Zhang , Y. Cheng , and G. Zhu . 2019. “The Complete Mitochondrial Genome Sequence and Phylogenetic Analysis for *Rhabdophis himalayanus* (Squamata: Colubridae).” Mitochondrial DNA Part B, Resources 4: 705–706. 10.1080/23802359.2024.2417975 PMC11494700 39439864

[ece374508-bib-0052] Yoshida, T. , R. Ujiie , A. H. Savitzky , et al. 2020. “Dramatic Dietary Shift Maintains Sequestered Toxins in Chemically Defended Snakes.” Proceedings of the National Academy of Sciences of the United States of America 117: 5964–5969. 10.1073/pnas.1919065117 PMC7084117 32094167

[ece374508-bib-0053] Zeller, G. , J. Tap , A. Y. Voigt , et al. 2014. “Potential of Fecal Microbiota for Early‐Stage Detection of Colorectal Cancer.” Molecular Systems Biology 10: 766. 10.15252/msb.20145645 PMC4299606 25432777

[ece374508-bib-0054] Zhang, J. , S. Tan , B. Lyu , et al. 2024. “Differences in Gut Microbial Composition and Characteristics Among Three Populations of the Bamboo Pitviper (*Viridovipera stejnegeri*).” Ecology and Evolution 14: e70742. 10.1002/ece3.70742 PMC11651729 39691431

[ece374508-bib-0055] Zhang, N. , L. Zhou , Z. Yang , and J. Gu . 2021. “Effects of Food Changes on Intestinal Bacterial Diversity of Wintering Hooded Cranes ( *Grus monacha* ).” Animals 11: 433. 10.3390/ani11020433 PMC7915383 33562375

[ece374508-bib-0056] Zhang, Z. , D. Xu , L. Wang , et al. 2016. “Convergent Evolution of Rumen Microbiomes in High‐Altitude Mammals.” Current Biology 26: 1873–1879. 10.1016/j.cub.2016.05.012 27321997

[ece374508-bib-0057] Zhao, G. , L. Zhou , Y. Dong , Y. Cheng , and Y. Song . 2017. “The Gut Microbiome of Hooded Cranes ( *Grus monacha* ) Wintering at Shengjin Lake, China.” MicrobiologyOpen 6: e00447. 10.1002/mbo3.447 PMC5458448 28127902

[ece374508-bib-0058] Zhao, J. , Y. Yao , D. Li , et al. 2018. “Characterization of the Gut Microbiota in Six Geographical Populations of Chinese Rhesus Macaques ( *Macaca mulatta* ), Implying an Adaptation to High‐Altitude Environment.” Microbial Ecology 76: 565–577. 10.1007/s00248-018-1146-8 29372281

[ece374508-bib-0059] Zhong, J. , K. Guo , Z.‐L. Liao , S.‐C. Hu , Y. Du , and X. Ji . 2022. “Comparative Analysis of the Gut Microbiota Composition Between Two Sea Snakes, *Hydrophis curtus*, and *Hydrophis cyanocinctus* .” Coral Reefs 41: 53–62.

[ece374508-bib-0060] Zhu, F. , K. Sun , H. Zhang , et al. 2024. “Comparative Analyses of *Lycodon rufozonatus* and *Lycodon rosozonatus* Gut Microbiota in Different Regions.” Ecology and Evolution 14: e70480. 10.1002/ece3.70480 PMC11495892 39440211

