# Supplementary material for: Characteristics of Microbiota in Different Segments of the Digestive Tract of Orange‐Collared Keelback (Rhabdophis himalayanus)
Source: Ecol Evol. 2026 Oct 9;16(10):e74508. doi: 10.1002/ece3.74508 (PMC13651714; doi:10.1002/ece3.74508)
Supplement: Supplementary file 1 [file ECE3-16-e74508-s001.pdf]

SampleID	Raw_Base(G)	Clean_Base(G)	Clean_Q20 (%)	Clean_Q30(%)	Clean_GC (%)	Effective (%)	NonHost_Base (G)
RH1.W	6.85	6.62	97.79	94.39	41.72	96.71	2.95
RH1.X	6.49	6.23	97.65	94.05	42.32	96.03	2.97
RH1.D	6.71	6.59	97.79	94.37	42.44	98.21	3.06
RH2.W	6.76	6.46	97.45	93.63	42.39	95.54	3.35
RH2.X	6.83	6.62	97.49	93.62	41.78	96.98	3.55
RH2.D	6.76	6.48	97.42	93.56	42.53	95.85	3.42
RH3.W	6.46	6.34	97.5	93.7	42.17	98.13	3.18
RH3.X	6.37	6.26	97.58	93.81	41.98	98.23	3.39
RH3.D	6.01	5.87	97.55	93.77	42.43	97.75	2.77
SD	0.28	0.24	0.14	0.32	0.30	1.08	0.26
Mean	6.58	6.39	97.58	93.88	42.20	97.05	3.18

Table S1. Statistical Information Sheet on Quality Control of Metagenomic Data

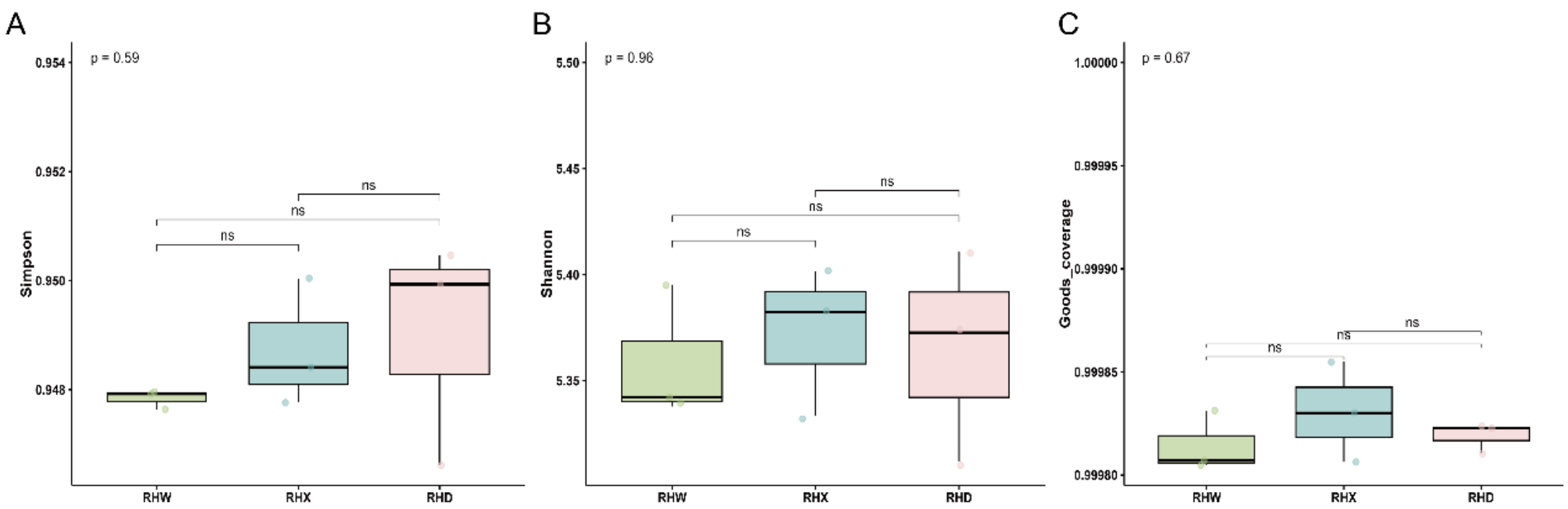

Figure S1. Kruskal-Wallis (KW) analysis of the alpha diversity of gut microbiota in three groups. Abbreviations: RHD, large intestine; RHX, small intestine; RHW, stomach; ns: $p > 0.05$.

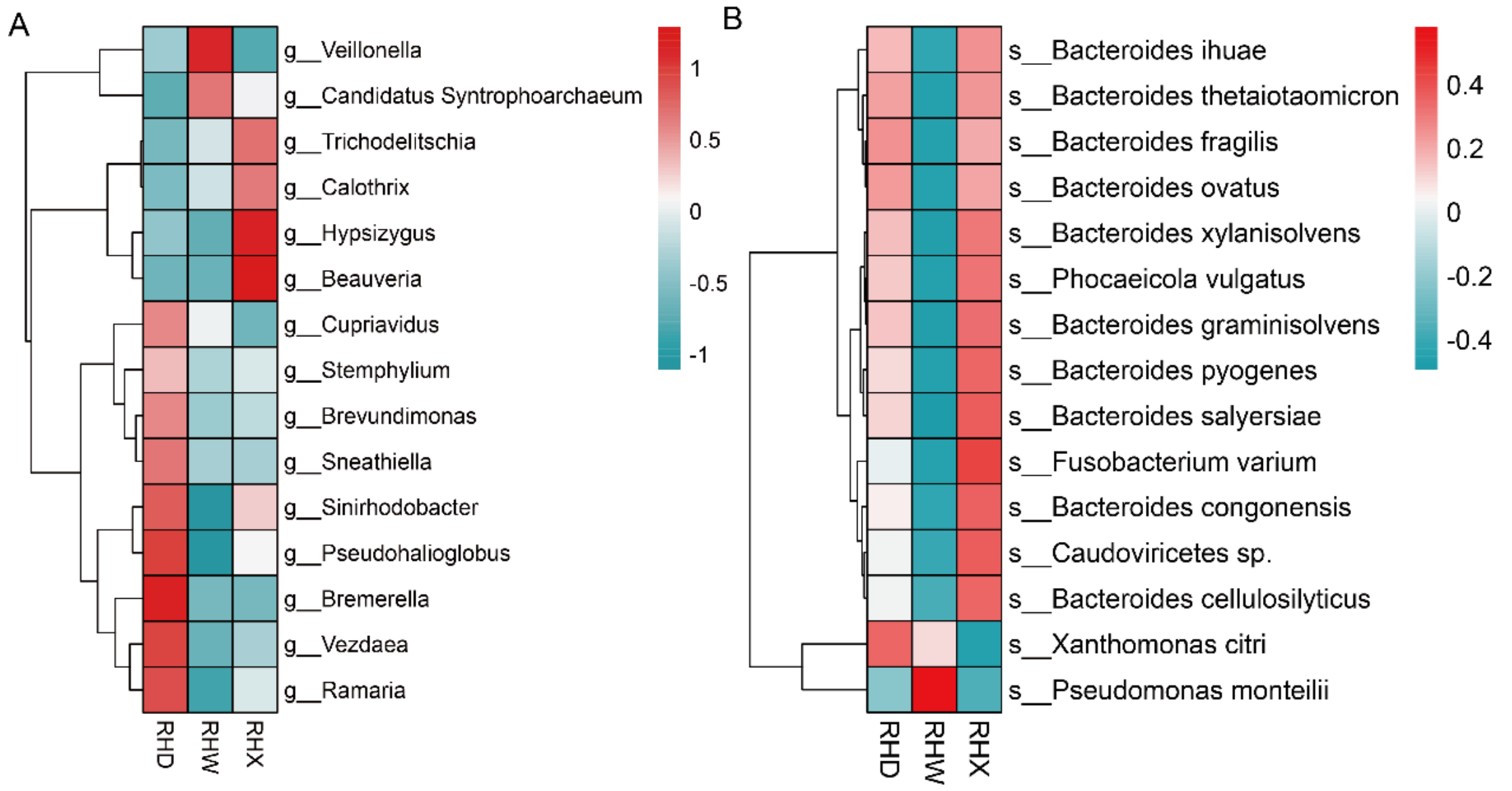

Figure S2. Differential abundance heatmap illustrating the top 15 genera (A) and species (B) with significant inter-group disparities identified via MetaStats analysis. Abbreviations: RHD, large intestine; RHX, small intestine; RHW, stomach.

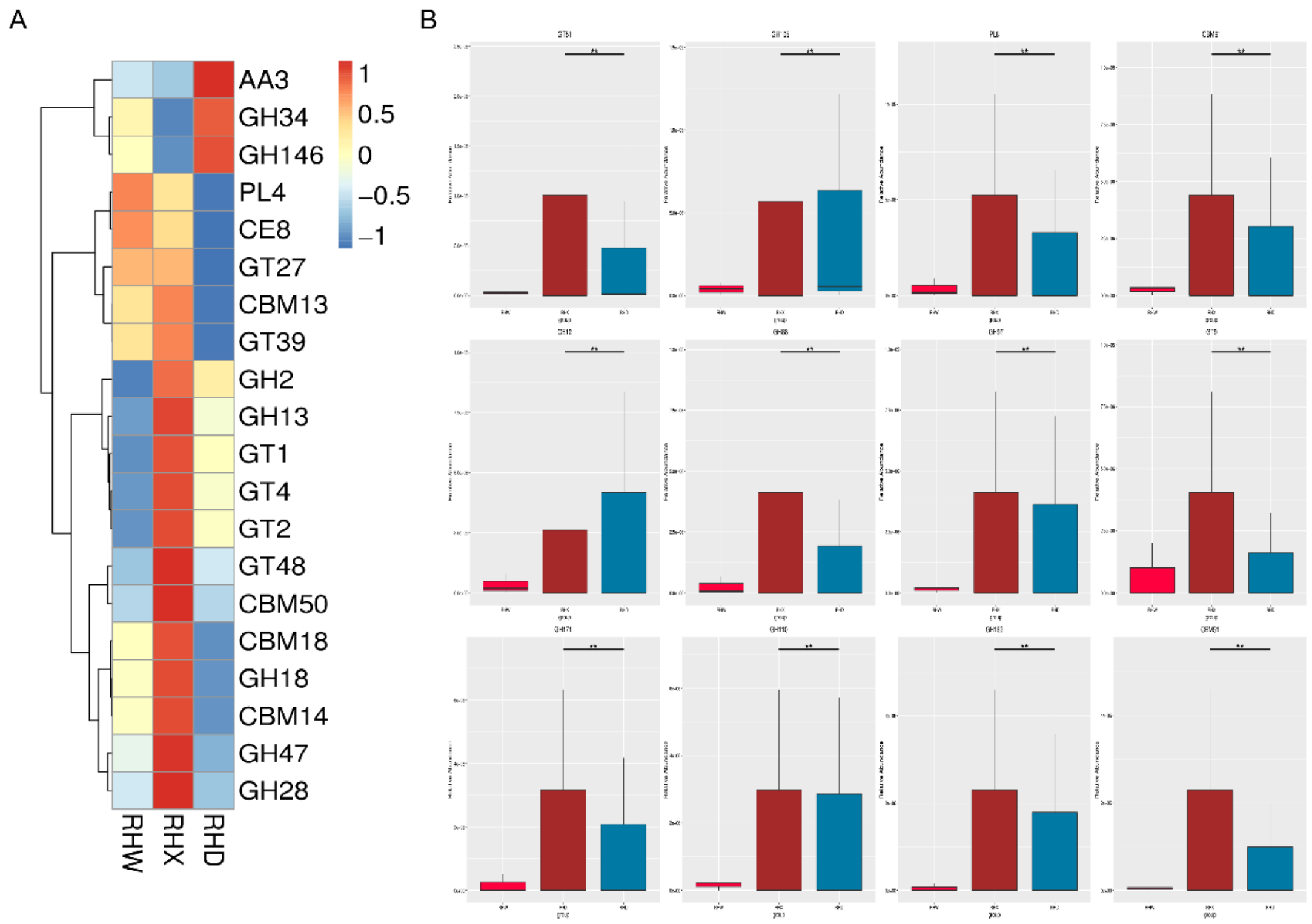

Figure S3. (A) Heatmap delineating inter-group compositional disparities among the top 15 most prevalent CAZy families at level 2 taxonomic hierarchy. (B) Differential abundance heatmap of the top 12 enzyme families with significant inter-group variations identified through MetaStats analysis, visualized at the CAZy level 2 classification. Abbreviations: RHD, large intestine; RHX, small intestine; RHW, stomach.
